# Testicular choriocarcinoma: a rare diagnosis in a patient presenting with hemorrhagic shock

**DOI:** 10.1093/jscr/rjad064

**Published:** 2023-02-22

**Authors:** Michael Mazzeo, Ratul Bhattacharyya, James Yang, Ida Molavi

**Affiliations:** School of Medicine, St. George’s University, GrenadaWest Indies; Department of Surgery, St. Joseph’s University Medical Center, Paterson, NJ, USA; Department of Surgery, St. Joseph’s University Medical Center, Paterson, NJ, USA; Department of Surgery, St. Joseph’s University Medical Center, Paterson, NJ, USA

## Abstract

Testicular choriocarcinoma is a rare and aggressive subtype of nonseminomatous germ cell tumors accounting for <1% of all germ cell tumors. We report an unusual case of testicular choriocarcinoma metastasis that presents as hemorrhagic shock. Diagnosis was unsuspected and difficult with vast other potential causes. This case highlights the importance of proper foundational workup and management that ultimately led to the appropriate definitive treatment of unusual manifestations of undiagnosed metastatic choriocarcinoma in a critical patient.

## INTRODUCTION

Choriocarcinoma is an uncommon type of a germ cell tumor. These neoplasms are trophoblastic in origin and are known to hematogenously metastasize to the lung, central nervous system and other genitourinary sites. Liver metastases is less common, occurring in 10% of patients with metastatic trophoblastic disease [1]. Testicular neoplasms is the most common solid organ malignancy in males between the ages of 15 and 35 and represent only 0.5–1% of all solid male cancers in the USA [2].

### Case report

**Figure 1:**
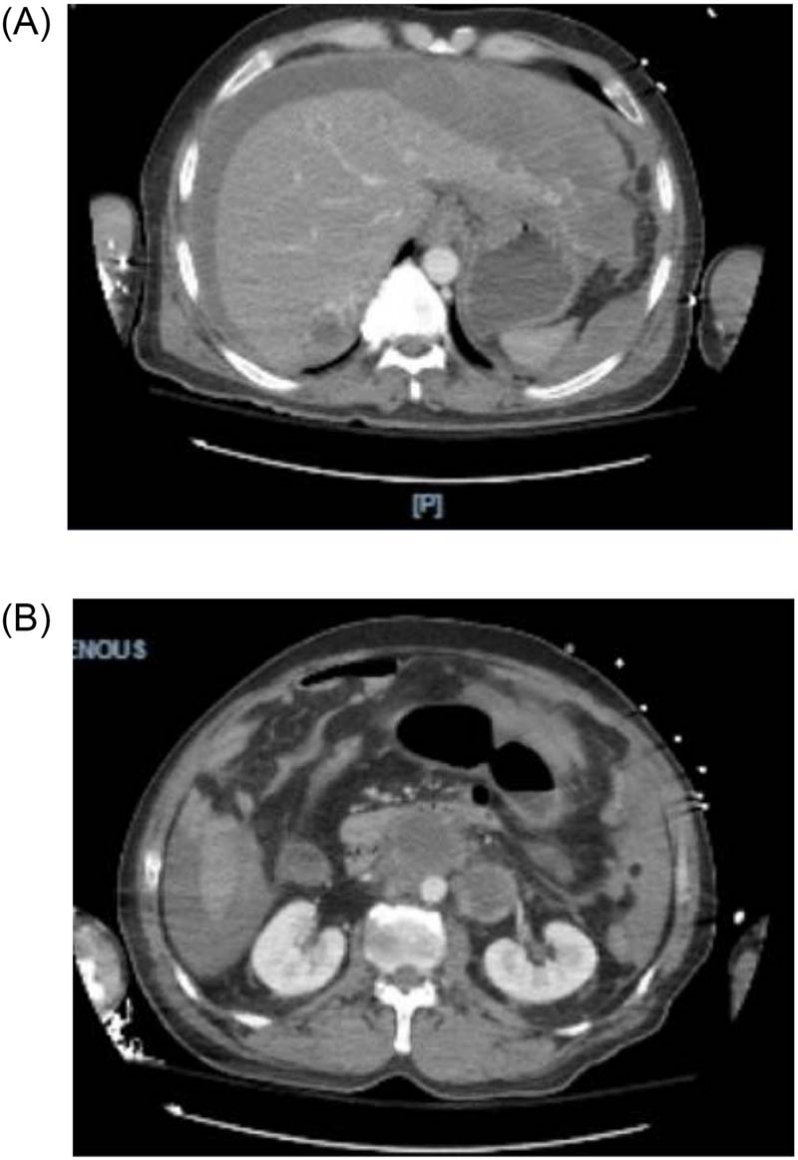
(**A, B**) CTA shows hemoperitoneum anterior to the left lobe of the liver.

A 44-year-old male with a past medical history of alcohol abuse presented to the emergency department with 2 days of right upper quadrant abdominal pain and vomiting. The patient was reported to have hematemesis prior to arrival. The patient was unable to give a comprehensive history due to altered mental status and was noted to be diaphoretic, pale and hypotensive on arrival. Hemoglobin, hbg, was noted to be 10.1 upon arrival; however, subsequent hbg was 5.3. Bedside arterial blood gas showed significant metabolic acidosis with a pH of 7.03 and lactic acid of 11. Massive transfusion protocol was started and vasopressor therapy was initiated. Bedside Focused Assessment with Sonography in Trauma, FAST, exam was performed and positive for fluid in the right upper quadrant. At this time, the patient had been stabilized and the decision was made to obtain a computed tomography angiography (CTA) of the chest, abdomen and pelvis. CTA revealed hemoperitoneum anterior to the left lobe of the liver, a large para-aortic and aortocaval mass with peripheral enhancement suggesting neoplastic adenopathy, measuring 5.2 × 8.1 cm, and a complex septated lesion in the right scrotum (Fig. 1A and B).

The patient was immediately taken from radiology to the operating room for exploratory laparotomy. Active bleeding was noted in the left lobe with lesions on the medial and lateral border of the left lateral segment. Argon beam coagulator was used to achieve hemostasis. An incisional biopsy of one of the lesions was performed.

**Figure 2:**
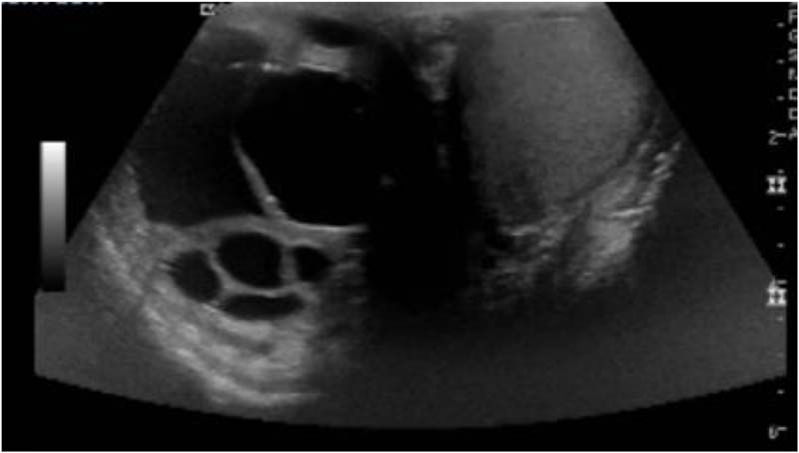
Right scrotum complex cystic mass.

**Figure 3:**
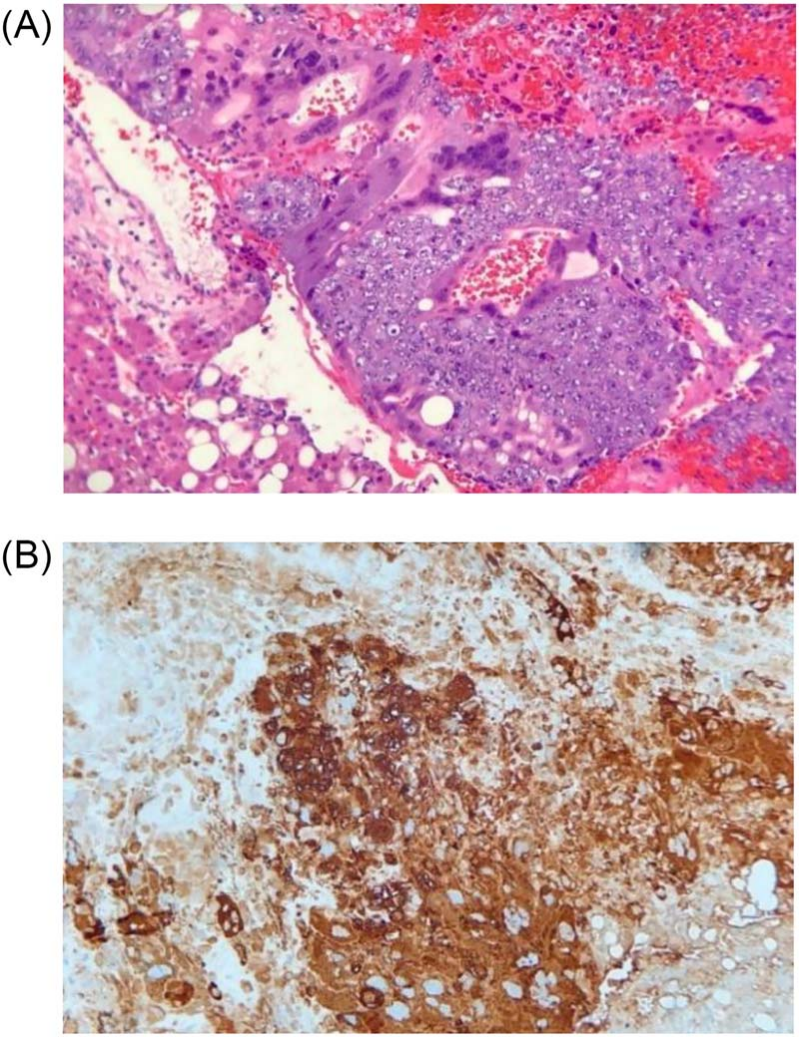
**(A)** Histologic section showing mononucleated trophoblast and multinucleated syncytiotrophoblast cells consistent with choriocarcinoma (Hematoxylin and eosin stain); **(B)** Immunohistochemistry for human chorionic gonadotropin (hCG) highlights tumors cells, which supports the diagnosis of choriocarcinoma (Immunohistochemical stain).

On postoperative Day 1, scrotal ultrasound was performed. The ultrasound findings were significant for a 6 cm complex cystic mass in the right scrotum (Fig. 2). Immunohistochemical analysis of the resected liver tumor showed positive reaction with pancytokeratin, HCG and SALL4 (Fig. 3A and B). Subsequent laboratory results showed an elevated b-hCG of 130, 985 mIU/ml, lactate dehydrogenase of 581 U/L and alpha fetoprotein of 2 ng/ml.

## DISCUSSION

Testicular cancers are categorized as either germ cell or non-germ cell tumors, with germ cell tumors further split between seminoma and non-seminomatous germ cell tumors. Choriocarcinoma is the rarest, comprising 1–3% of all testicular neoplasms [2]. Our patient’s initial clinical picture was convoluted as the patient had also presented with hematemesis and history of alcohol abuse. Given this history, upper gastrointestinal bleed had been the initial top differential diagnosis.

In a patient presenting with hemorrhagic shock, suggestive of intra-abdominal hemorrhage, it was pertinent to consider causes such as trauma, rupture of diseased blood vessels, hemorrhagic pancreatitis and vascular tumors. Rupture of metastatic hepatic tumors is rarer due to their fibrotic and less vascular nature; choriocarcinoma is an exception due to being a primarily vascular and hemorrhagic tumor with a propensity to rupture.

Possible mechanisms of rupture that have been proposed include direct pressure by the tumor against the capsular surface due to increased intra-abdominal pressure or increased intravascular pressure secondary to tumor emboli, resulting in intra hepatic venous obstruction [3, 4].

## CONCLUSION

Hemorrhagic shock is a critical finding with a vast differential diagnosis. The key to management is prompt recognition and proper treatment. In this case, the clinical presentation of choriocarcinoma was subtle as CT imaging depicting multiple enhancing masses in the liver and lungs cannot be easily differentiated from other hyper vascular tumors [5]. In the setting of hemorrhagic shock, volume repletion with blood products and use of vasopressor therapy must be quickly initiated. Once stabilized, each subjective and objective finding is important in leading to the correct diagnosis and management.
